# Hedgehog and *Drosophila* germ cell migration

**DOI:** 10.1242/dev.205291

**Published:** 2025-12-19

**Authors:** Girish Deshpande, Ji Hoon Kim, Caitlin D. Hanlon, Paul Schedl, Deborah J. Andrew

**Affiliations:** ^1^ Department of Molecular Biology, 119 Lewis Thomas Laboratory, Washington Road, Princeton University, Princeton, NJ 08540, USA; ^2^ Visiting Faculty, Department of Biology, Indian Institute of Science Education and Research, Pashan, Pune, Maharashtra 411008, India; ^3^ Department of Cell Biology, Johns Hopkins University School of Medicine, 725 N. Wolfe St., Baltimore, MD 21205, USA; ^4^ Department of Biological Sciences, Quinnipiac University, 275 Mount Carmel Avenue, Hamden, CT 06518, USA

**Keywords:** *Drosophila*, Germ cells, Primordial germ cells, Somatic gonadal precursors, Hedgehog

## Abstract

The functional gonads, essential for the continuity of a species, have both somatic and germline components. Newly formed germ cells are quiescent and are often physically isolated from the rest of the soma, protecting them from the signals that control somatic specification and differentiation. Nonetheless, the sequestered germ cells must ultimately navigate through the embryo to meet up with the somatic gonadal components. Forward genetic screens conducted in *Drosophila* have uncovered several crucial factors that generate both attractive and repulsive signals controlling germ cell movement. Efforts to reveal how the range of molecular players coordinate their activities to ensure that navigation is a robust and reproducible process have led to exciting, albeit sometimes contentious, discoveries. Herein, we summarize evidence for Hedgehog functioning in a single pathway from the signal source to signal reception to the downstream cytoskeletal events controlling the directed movement of germ cells to the site of gonad formation.

## Teaser

**Summary:** This Review discusses how, in *Drosophila* embryos, the morphogenetic signaling ligand Hedgehog moonlights as a chemoattractant for migrating germ cells via a noncanonical signal transduction pathway to direct cell motility.

## Introduction

‘It's all one song’

Neil Young, 1996 Crazy Horse Tour

The *Drosophila* gonad comprises germline and somatic components that arise at distant embryonic positions and require directed cell migration to reach their shared destination to coalesce. The germline contribution comes from the primordial germ cells (PGCs), which form at the posterior embryonic pole and are the first cells in the embryo to form (Hegner, 1914; Huettner, 1923). *Drosophila* embryonic development ensues post-fertilization when the male and female pronuclei fuse to give rise to a single-cell embryo. Rather than undergoing typical cytokinesis, the single nucleus undergoes 13 rapid serial nuclear division cycles resulting in a multinuclear syncytium. Although almost all nuclei will eventually migrate to the periphery and form cells, a few nuclei, along with their associated centrosomes, precociously migrate to the posterior pole and into the maternally deposited and posteriorly anchored specialized cytoplasm known as pole plasm. The arrival of these nuclei leads to formation of bud protrusions, enriched for the pole plasm contents, which subsequently pinch off and form the early pole cells or PGCs (Kilwein et al., 2025). Both the formation and proper specification of the PGCs ensure germline/soma segregation, and are largely controlled by maternal determinants (Boswell and Mahowald, 1985; Ephrussi et al., 1991; Illmensee and Mahowald, 1974; Mahowald, 1962). The somatic gonadal precursors (SGPs), which arise later in development (Riechmann et al., 1998), are derived from lateral mesoderm in posterior parasegments (Clark et al., 2007; DeFalco et al., 2003). The formation and function of SGPs require localized Hox activity in combination with input from both segment polarity and dorsal-ventral patterning genes (Boyle et al., 1997; Boyle and DiNardo, 1995; Greig and Akam, 1995; Moore et al., 1998a,b; Riechmann et al., 1998).

**Fig. 1.**
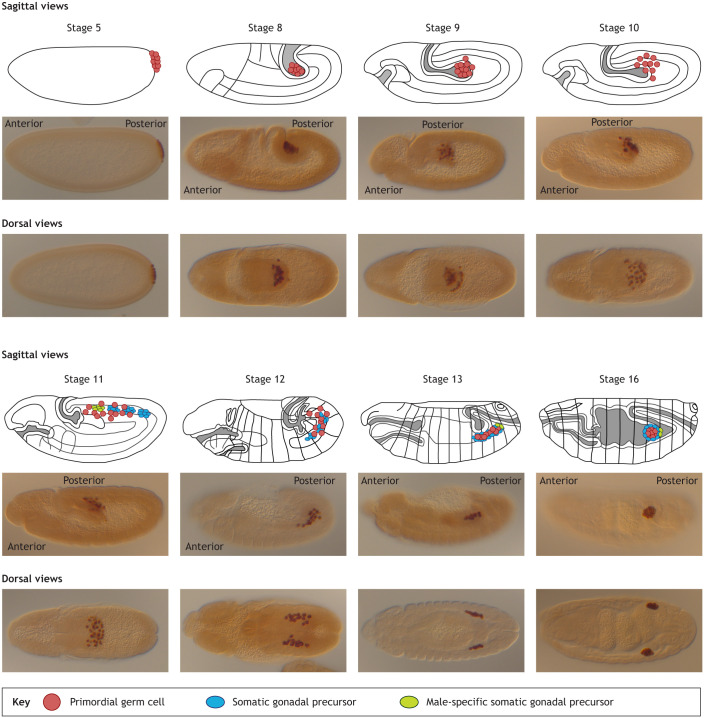
**The migratory journey of primordial germ cells (PGCs).** Cartoons are sagittal views with both PGCs (red) and somatic gonadal precursors (SGPs; blue/green) indicated. Embryos below each cartoon image are wild-type embryos of the corresponding stage. PGCs form early during embryonic stage 5 and are passively brought into the interior of the embryo by the movements of gastrulation and germ band elongation. By stage 8, the posterior portion of the germband is extended dorsally and folded interiorly to create a blind-ended pocket of posterior midgut endoderm containing the PGCs. Subsequently, PGCs individualize and migrate dorsally through the midgut endoderm epithelium (stage 9/10). They then split into two similarly sized lateral cell clusters and begin migrating in an antero-lateral direction (stage 11/12) through mesoderm and toward the SGPs found on either side of the embryo in parasegments 10-12 (also 13 in males). Soon after PGCs and SGPs contact and intermingle, they compact to form the primitive embryonic gonad in the fifth abdominal segment (late stage 13 to stage 15) (Callaini et al., 1995; Jaglarz and Howard, 1995; Warrior, 1994). For immunostaining, embryos were fixed with 4% formaldehyde in heptane, devitellinized with methanol, and subsequently stained with a 1:100 dilution of anti-Vasa (PGC marker) (Santa Cruz Biotechnology, sc-30210, RRID:AB_793874) in PBS). Goat anti-rabbit biotinylated secondary antibody was used at a dilution of 1:1000 (Thermo Fisher Scientific, A16108), followed by signal magnification and detection using horseradish peroxidase using the VECTASTAIN ABC kit (Vector Laboratories, PK4000). Sagittal views on top, dorsal views below.

Bringing together the germline and somatic components of the gonad begins with the major cell movements of gastrulation and germ band elongation, which carry the PGCs into the blind (closed) end of the posterior endoderm pocket (Fig. 1). From there, individual PGCs migrate dorsally through the epithelial layer of posterior midgut endoderm. Once across the gut endoderm, PGCs split into two lateral clusters and continue to migrate as individual cells through the mesoderm to meet up with the SGPs, which at this stage are found in two bilateral clusters spanning parasegments 10-13. Upon reaching the SGP clusters, PGCs continue to migrate anteriorly to distribute across the SGP clusters. After the PGCs and SGPs have coalesced, they undergo compaction to form two symmetrically positioned spherical gonads in the lateral regions of the fifth abdominal segment (Fig. 1) (Boyle and DiNardo, 1995; Jemc, 2011; Rabinowitz, 1941; Sonnenblick, 1941). Although SGP clusters can migrate toward each other and undergo normal compaction in the absence of PGCs (Clark et al., 2007), the reverse is not true; PGC navigation absolutely requires SGPs (Boyle et al., 1997; Moore et al., 1998b).

Key questions in the field of gonad formation focus on identifying the attractant(s) SGPs produce to guide migrating PGCs to the site of gonadal coalescence and to understand how reception of this attractant is translated into directed cell movement. In the following sections, we present evidence for the identity of this cue and the mechanism underlying navigation toward the source of the cue. Based on the principles of developmental biology, the guidance system should fulfill several important criteria: (1) the attractant(s) should be produced by the cells that are guiding PGC migration (i.e. the SGPs); (2) the attractant must reach the migrating cells; (3) the receptor(s) for the attractant should be expressed in the migrating PGCs; (4) reception of the attractant signal should translate into directed cell movement. The work described below demonstrates that the signaling ligand Hedgehog (Hh) fulfills these criteria and allows us to put forward a testable model to resolve any remaining inconsistencies.

## Hmgcr: an enzyme required for PGC attraction

SGPs and other surrounding tissues provide attractive and repulsive guidance cues for PGC navigation. Indeed, early studies by Van Doren and colleagues revealed that 3-hydroxy-3-methylglutaryl-CoA reductase (Hmgcr), a rate-limiting enzyme in the mevalonate biosynthetic pathway (Box 1), regulates the production and/or activity of a chemoattractant derived from SGPs to guide PGCs (Van Doren et al., 1998) (Table 1). In embryos mutant for *Hmgcr*, many PGCs fail to exit the endoderm and those that do scatter widely throughout the embryo, failing to associate with the SGPs (Van Doren et al., 1998). Moreover, ectopic expression of Hmgcr in a wide variety of tissues, including the epidermis and central nervous system (CNS), redirects a large subset of migrating PGCs to those tissues (Kenwrick et al., 2019; Van Doren et al., 1998). Further supporting the idea that *Hmgcr* plays a key role in synthesis or transmission of the SGP attractant, *Hmgcr* has a dynamic pattern of expression. Initially, it is expressed broadly in the mesoderm, but as the embryo develops *Hmgcr* expression becomes progressively restricted, first to the posterior parasegments and ultimately to only the cells that become the SGPs (Santos and Lehmann, 2004; Van Doren et al., 1998).

Box 1. The mevalonate biochemical pathway

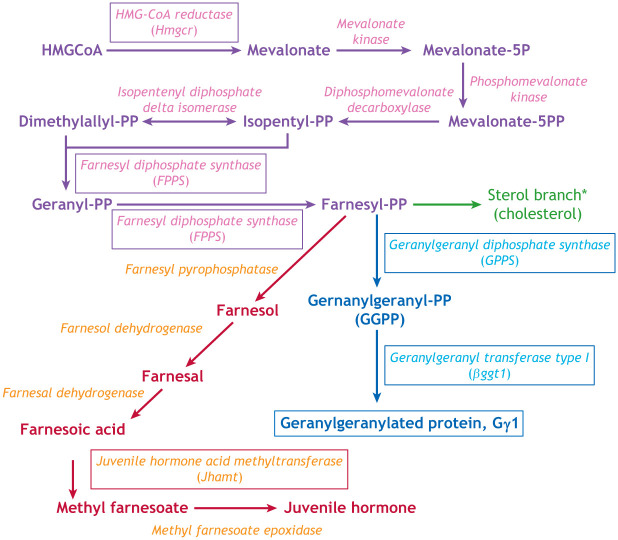

Shown is the isoprenoid (purple), sterol (green), prenylation (blue) and juvenile hormone (red) biosynthetic pathway in insects (Buhaescu and Izzedine, 2007; Noriega, 2014; Santos and Lehmann, 2004). Metabolites are shown in bold, the enzymes in italics. Mutations in the genes encoding the enzymes that are boxed have been demonstrated to lead to primordial germ cell (PGC) mismigration. Note that insects do not produce most of the enzymes in the sterol branch of the pathway (asterisk), which leads to cholesterol production and must obtain cholesterol from dietary sources.

**Table 1.** Genes affecting primordial germ cell (PGC) migration

Gene	Biochemical function in PGC navigation	References for the role of gene in PGC navigation
*HMG coenzyme A reductase* (*Hmgcr*)	Rate-limiting enzyme in the mevalonate pathway (isoprenoids and cholesterol); expressed in SGPs and earlier mesoderm; release and transport of Hh	Deshpande and Schedl, 2005; Kenwrick et al., 2019; Van Doren et al., 1998
*Farnesyl pyrophosphate synthase* (*Fpps*)	Farnesyl diphosphate synthase – generates geranyl-PP and farnesyl-PP downstream of Hmgcr	Santos and Lehmann, 2004
*quemao* (*qm*)	Generates geranylgeranyl-PP downstream of Hmgcr	Santos and Lehmann, 2004
*Geranylgeranyl transferase type I* (*βggt-I*)	Geranylgeranyl transferase type I-transfers geranylgeranyl groups to proteins	Santos and Lehmann, 2004
*juvenile hormone acid methyltransferase* (*jhamt*)	Juvenile hormone acid methyl transferase – enzyme downstream of the farnesyl pyrophosphate branch of the isoprenoid biosynthesis pathway	Barton et al., 2024
*hedgehog* (*Hh*)	Attractive ligand secreted by SGPs; also expressed and required in other mesoderm and ectoderm tissues	Deshpande et al., 2001, 2023; Deshpande and Schedl, 2005; Kim et al., 2021
*Gγ1*	Downstream target for Hmgcr-isoprenoid biosynthesis pathway; Hh intracellular trafficking	Deshpande et al., 2009
*dispatched* (*disp*)	Assembly and sorting of large Hh punctae	Gallet et al., 2003
*Multi-drug resistance 49* (*Mdr49*)	Source of cholesterol for Hh processing and downstream signaling	Deshpande et al., 2016
*Niemann-Pick type C-1a* (*Npc1a*)	Source of cholesterol for Hh processing and downstream signaling	Bialistoky et al., 2019
*tout velu* (*ttv*)	Regulator of heparan sulfate proteoglycan biosynthesis; required for long-range Hh transport	Deshpande et al., 2007
*shifted* (*shf*)	Hh stability and diffusion; long-distance transmission of Hh	Deshpande et al., 2013
*patched* (*ptc*)	Inhibitor of Hh signaling, blocks Smo activity in the absence of Hh	Deshpande et al., 2001; Kim et al., 2021
*Protein kinase A - C1* (*Pka-C1*)	Phosphorylation and activation of Smo downstream of Hh	Deshpande et al., 2001
*fused* (*fu*)	Smo stabilization at membrane	Deshpande et al., 2001
*smoothened* (*smo*)	Positive effector of Hh signaling – localization of Tre1 to plasma membrane	Deshpande et al., 2001; Kim et al., 2021
*Trapped in endoderm 1* (*Tre1*)	GPCR required for germ cell navigation downstream of Hh signaling through Smo	Kamps et al., 2010; Kim et al., 2021; Kunwar et al., 2003, 2008; LeBlanc and Lehmann, 2017; Lin et al., 2020
*PIP5K59B* (*dPIP5K*)	Enzyme recruited by Tre1 that generates PIP(4,5)P2 at migration front	Kim et al., 2021
*Wiscott-Aldrich Syndrome protein* (*WASp*)	Activated by PIP(4,5)P2; recruits Arp2,3 to PGC migration front to drive actin assembly	Kim et al., 2021

Note that loss-of-function mutations in each of the corresponding genes leads to the same phenotype: a failure of PGCs to reach the gonad.

GPCR, G protein-coupled receptor Hh, Hedgehog; SGP, somatic germ cell progenitors.

In mammals, Hmgcr is an enzyme upstream of both the isoprenoid and cholesterol biosynthetic pathways. Flies lack the genes encoding most enzymes that function in the cholesterol branch and consequently must obtain cholesterol in their diet (Clark and Bloch, 1959; Clayton, 1964); therefore, it is the production of isoprenoids that is relevant to PGC migration (Box 1). Supporting this idea, loss of two downstream biosynthetic enzymes, farnesyl-pyrophosphate synthase (FPPS) and geranylgeranyl pyrophosphate synthase (GPPS), required to produce the isoprenoid geranylgeranyl pyrophosphate (GGPP), as well as loss of the enzyme that transfers GGPP to protein substrates, geranylgeranyl transferase type I (βggt1), result in PGC migration defects (Santos and Lehmann, 2004) (Table 1; Box 1). These findings indicate that Hmgcr functions in PGC migration through its rate-limiting production of the precursors required for geranylgeranylation of a protein, which either acts as the attractant itself, or is required for the production, release or transport of the attractant.

## A candidate attractant: Juvenile Hormone

A recent study suggests that Juvenile Hormone (JH) is the PGC attractant produced downstream of Hmgcr (Barton et al., 2024) (Table 1). Both loss and misexpression of the gene encoding Juvenile hormone acid O-methyltransferase (Jhamt), the enzyme that catalyzes a late step in JH production, cause mild defects in PGC migration. Notably, JH is produced in a different branch of the isoprenoid biosynthetic pathway (Box 1), which leads to the production of farnesol, farnesal and farnesoic acid downstream of farnesyl pyrophosphate (FPP) (Noriega, 2014), not the production of GGPP previously implicated in PGC migration by the same group (Santos and Lehmann, 2004).

The discovery that mutations in enzymes functioning in two independent branches of the isoprenoid pathway affect PGC migration, as well as the more severe migration defects observed with loss of *Hmgcr*, suggests that Hmgcr could act through two distinct downstream effectors. However, the data supporting a role for JH are less clear. First, although *jhamt* transcripts are observed in mesoderm, there is no direct evidence supporting *jhamt* expression in the SGPs, the known source for the PGC attractant (Barton et al., 2024; Halfon et al., 2011; Niwa et al., 2008). Second, the reporter used for JH pathway activation is not obviously detectable in PGCs; the highlighted PGC staining is not above the background seen in nearby ventral cells and is much below the levels observed in the more dorsal mesodermal cells (Barton et al., 2024). Furthermore, *jhamt* expression is limited across the embryo (Barton et al., 2024; Halfon et al., 2011; Niwa et al., 2008), which contrasts with the observation that ectopic expression of Hmgcr in a variety of tissues (Kenwrick et al., 2019; Van Doren et al., 1998), including both the epidermis and CNS, tissues that do not normally express *jhamt*, is sufficient to attract PGCs (Kenwrick et al., 2019; Van Doren et al., 1998). Arguably, these observations may improve over time as more tools/reagents become available; as it stands, however, stronger evidence is needed to support a direct role for JH in PGC navigation.

## A candidate attractant: Hedgehog

**Fig. 2.**
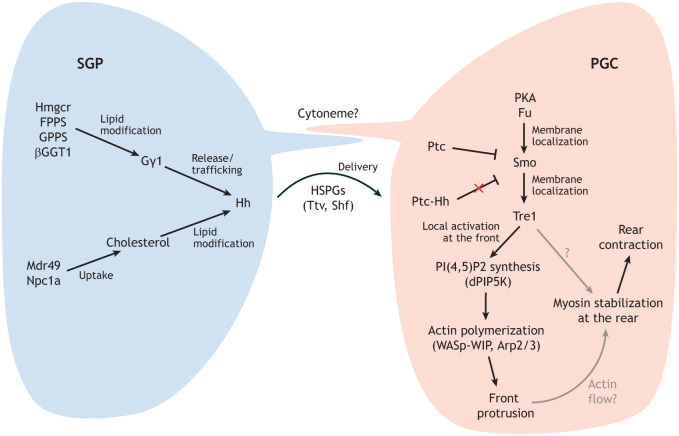
**Signaling from the somatic gonadal precursors (SGPs) to the primordial germ cells (PGCs) to control PGC navigation.** Model of how the Hedgehog (Hh) signal, produced initially in mesoderm and then specifically in SGPs, is processed, released and transmitted to PGCs to guide their migration to the gonad. In SGPs, the isoprenoid branch of the mevalonate pathway, in which the rate-limiting enzyme is Hmgcr (Box 1), produces geranylgeranylated Gγ1, which is required for Hh release and transport from Hh-producing cells. Cholesterol (obtained in the diet and taken up by the cholesterol transporters Mdr49 and Npc1a) modification of Hh is essential for signaling function. Heparin sulfate proteoglycans (HSPGs) and the enzymes required for their synthesis are required for Hh transport, which is proposed to be through cytonemes present on both the SGP signal-sending cells and the PGC signal-receiving cells. In PGCs, Hh-bound Ptc is internalized and Smo translocates to the plasma membrane at the site of signal activation, also recruiting the Tre1 receptor. Tre1 through dPIP5K-dependent synthesis of PI(4,5)P2 activates WASp, which in turn activates the Arp2,3 complex to create a branched actin protrusion at the migration front. This branched actin network defines the front of the cell and localizes and stabilizes the intrinsic actin flows and MyoII localization to the rear of the cell.

Data reported by our groups over the past quarter century provide strong evidence that the secreted protein Hedgehog (Hh) (reviewed by Ingham, 2022; Zhang and Beachy, 2023) acts downstream of Hmgcr (Table 1; Fig. 2). Importantly, Hh is expressed in SGPs and their precursors (Deshpande et al., 2001). Hh is also broadly expressed in both ectoderm and other mesoderm, in keeping with the observation that ectopic expression of Hmgcr in a wide variety of tissues can attract PGCs to those tissues.

Null mutations in *hh* severely disrupt PGC navigation, even before SGP specification, when both Hh and Hmgcr are more broadly expressed in mesoderm (Deshpande et al., 2001; Kim et al., 2021; Moore et al., 1998b; Van Doren et al., 1998). Additionally, ectopic expression of Hh using an enhancer mutant allele of *hh* (Perez et al., 2011) or the Gal4:UAS system cause PGC migration defects without obvious defects in embryonic patterning or SGP specification, which suggests a PGC-specific response (Deshpande and Schedl, 2005; Deshpande et al., 2001, 2013; Kim et al., 2021). Moreover, the effects of ectopic expression of Hmgcr on PGC migration can be substantially mitigated by knocking down Hh in the same cells using RNA interference, placing Hh downstream of Hmgcr in the same developmental pathway (Deshpande et al., 2023).

In the sections below, we elaborate on how Hmgcr plays a crucial role in Hh transmission, as well as how canonical downstream Hh signaling components are expressed in the PGCs and are required for these cells to navigate effectively to the gonad (Deshpande et al., 2001, 2023; Kim et al., 2021).

## Transmission of Hh to migrating cells

### Linking Hmgcr to Hh secretion

Hh is expressed broadly in the early embryo, including in mesoderm that does not attract PGCs. So, how is the Hh signal produced by SGPs specific for attracting PGCs? Evidence suggests that Hmgcr confers specificity by controlling the release and long-range activity of Hh. For example, we have shown that Hmgcr promotes more efficient Hh transmission when co-expressed in Hh-producing cells compared to when Hmgcr is expressed in adjacent cells lacking Hh production (Deshpande and Schedl, 2005). Also, in *Hmgcr* mutant embryos, Hh protein accumulates in puncta in the Hh-sending ectodermal cells, disrupting Hh signaling to neighboring cells. Consequently, Smoothened (Smo) relocalization to the membrane – an essential step in Hh signaling – fails, and the expression of Wingless (Wg), which depends upon the reception of the Hh signal, is not sustained. These findings suggest that Hmgcr is involved in modulating the range or strength of the Hh signal. Correspondingly, the PGC mismigration that is observed with ectopic expression of Hmgcr in the CNS is mitigated when Hh function is simultaneously knocked down in the same cells. This suggests that the PGC attractant activity of Hmgcr is Hh dependent, placing Hh downstream of Hmgcr (Deshpande et al., 2023).These findings indicate that an important target of the Hmgcr biosynthetic pathway for PGC migration is either the Hh protein or a factor crucial for Hh release and/or trafficking.

Hh release may require a geranylgeranylated protein because PGC migration defects are observed when other enzymes needed for the production and attachment of the isoprenoid GGPP are lost (Santos and Lehmann, 2004). The direct geranylgeranylation of Hh has not been reported; therefore, the most likely target is a factor(s) required for Hh release or transport. An excellent candidate in this regard is a subunit of the heterotrimeric G protein Gγ1, which functions in the transport of cargo from the trans-Golgi network to the plasma membrane. During embryonic heart development, the activity of this heterotrimeric complex requires Hmgcr-dependent geranylgeranylation of the Gγ1 subunit (Yi et al., 2006). Indeed, in embryos mutant for zygotic *Gγ1* function, Hh protein accumulates in *hh*-expressing cells, instead of being released (Deshpande et al., 2009) (Table 1). Similar effects on Hh release have been observed with mutations in *quemao* (*qm*), which encodes GPPS. Mutations in *G*γ*1* genetically interact with loss-of-function mutations in *Hmgcr* and *hh* to disrupt PGC navigation, consistent with the idea that *G*γ*1* participates in the same PGC migration pathway. The connection between Gγ1 and *Hmgcr* in PGC migration is further supported by two independent observations. First, mesodermal expression of a dominant-negative *Gγ1* mutant that cannot be geranylgeranylated disrupts PGC migration. Second, heterozygosity for either of two different *Gγ1* loss-of-function alleles suppresses the PGC migration defects induced by ectopic expression of *Hmgcr* in the CNS (Deshpande et al., 2009).

### Manipulating other signaling components required for Hh long-range signaling

Full Hh function in all bilaterians requires proteolytic cleavage and the covalent attachment of both cholesterol and palmitate to an N-terminal fragment of the protein (Buglino and Resh, 2012; Siebold and Rohatgi, 2023). These lipid modifications allow Hh to be embedded into the outer leaflet of the plasma membrane and assembled into large punctate structures for long-distance signaling, as well as sorting within the Hh-producing cell by the cholesterol transporter Dispatched (Disp) (Gallet et al., 2003). Cholesterol modification is also required for Hh binding to the Patched (Ptc) receptor. Indeed, multiple steps in Hh signaling involve cholesterol trafficking (Hu and Song, 2019). Flies and other insects do not synthesize cholesterol and instead rely on dietary sources for this lipid (Clark and Bloch, 1959; Clayton, 1964). Consistent with a role for Hh in *Drosophila* PGC navigation, mutations in genes that encode the transporters required for cholesterol uptake, *Mdr49* and *Npc1a*, both alone and in transheterozygous combination, cause migration defects with many PGCs failing to reach the gonad (Bialistoky et al., 2019; Deshpande et al., 2016) (Table 1). Importantly, these migration defects can be rescued by providing the mothers with a high-cholesterol diet (Deshpande et al., 2016).

Two other factors implicated in Hh ligand distribution disrupt PGC navigation when mutated: *tout velu* (*ttv*) and *shifted* (*shf*) (Deshpande et al., 2007, 2013) (Table 1). Ttv is a glycosyltransferase that initiates the synthesis of heparan sulfate (Leisico et al., 2022), which is covalently attached to core extracellular matrix proteins to form heparan sulfate proteoglycans (HSPGs). HSPGs are involved in the long-distance transport of Hh (Han et al., 2004). In addition, Shf stabilizes the interaction between Hh and HSPGs, which further allows Hh ligands to be transmitted over longer distances (Gorfinkiel et al., 2005).

### A role for cytonemes in Hh transmission?

The cellular mechanism for delivery of the active Hh ligand from SGPs to PGCs remains unclear. The lipid modifications on the active N-terminal Hh signal (e.g. cholesterol and palmitate) indicate that Hh is associated with vesicles or the plasma membrane. Indeed, Hh signaling in other tissues is transmitted by cytonemes (Bischoff et al., 2013; Chen et al., 2017), thin filopodia-like actin-rich cellular protrusions that allow for direct long-range, cell-to-cell communication (Kornberg and Roy, 2014). Such structures would provide an optimal mechanism for delivery of Hh signal across the midgut endoderm and would fit with the distances over which the attractant is proposed to function (Kenwrick et al., 2019). Moreover, a guidance mechanism that uses cytoneme extensions for sending Hh signals from the SGPs to the PGCs would help ensure that reception of the Hh signal would be polarized, targeting the leading edge of the migrating PGCs. This would provide a more plausible mechanism for translating a signal from a distant source (spread over several parasegments) into directed migration than would a diffusion-based gradient of secreted Hh ligand.

Altogether, finding that mutations disrupting pathways required for (1) Hh release (components in the mevalonate pathway: Hmgcr, FPPS, GPPS, βggt1 and Gγ1), (2) generation of a functional Hh signal (cholesterol transporters), and (3) long-distance Hh transport (enzymes required for HSPG production) affect PGC migration further implicate Hh in this process as an attractant signal provided by SGP cells.

## PGCs detect Hedgehog signaling

Since in many contexts Hh functions as a morphogen and can impact cell fate decisions, manipulating *hh* activity (e.g. through the ectopic expression or mutation of *hh* or genes required for Hh transmission) could impact PGC migration indirectly by altering cell fates. However, if genes that function cell-autonomously in the reception of Hh signals are required in PGCs, this would firmly link the Hh signaling pathway to their migration towards the SGPs. This is indeed the case: both loss and overexpression of cell-autonomous Hh signaling components also disrupt PGC navigation (Table 1). Importantly, PGCs are transcriptionally silent until the stages when they migrate through the midgut primordia on their way to the SGPs (Lebedeva et al., 2018) and thus rely on maternally encoded transcripts for function. Consistent with the delayed transcriptional activation of zygotic genes, we find that maternal loss of *smo*, a key positive effector downstream of Hh signaling detection, results in inappropriate spreading of PGCs throughout the posterior region (Deshpande et al., 2001; Kim et al., 2021), even when the somatic function of *smo* is provided paternally and embryonic patterning and SGP specification are normal (Deshpande et al., 2001). Likewise, maternal loss of *Protein kinase A - C1* (*Pka-C1*) or of *fused* (*fu*), which encode factors crucial for Smo relocalization to the plasma membrane upon pathway activation (Claret et al., 2007; Jia et al., 2004; Jiang and Jia, 2015; Li et al., 2014), lead to consistent defects in PGC migration (Deshpande et al., 2001, 2023). Finally, maternal loss of the Hh receptor gene *ptc* leads to clumping of PGCs (Deshpande et al., 2001), whereas Ptc overexpression in PGCs leads to their mismigration (Kim et al., 2021). Together, these studies reveal that Hh downstream signaling machinery is required in PGCs for their directed migration and that Hh directly activates the pathway in these cells.

Early evidence arguing against Hh functioning as the SGP attractant was that key downstream Hh signaling components (Ptc and Smo) and a reporter of Hh activation and downstream transcriptional events (a *ptc*-*lacZ* fusion transgene) could not be detected in PGCs (Renault et al., 2009). However, with better tools and higher resolution microscopy, Kim and colleagues have demonstrated that both Hh and its downstream components (Ptc and Smo) are localized as expected if Hh functions as the signal (Kim et al., 2021). Using a Bac construct encoding a functional GFP-tagged version of Hh (Chen et al., 2017), Kim and co-workers have observed Hh-GFP protein in SGPs, as previously reported, but have also found high levels of Hh-GFP in large intracellular puncta within PGCs, consistent with Hh protein internalization following binding to the receptor and activation of the pathway. Indeed, these Hh puncta are observed in PGCs during their migration across the endoderm pocket (stage 9) through gonadal coalescence (stage 14). Endogenous Ptc has also been detected in PGCs, as well as the Ptc-*lacZ* reporter of transcriptional events downstream of Hh, indicating that Hh signaling is active in PGCs. Importantly, both endogenous Smo and Smo-GFP (expressed in PGCs using a germline-specific Gal4 line) colocalize with actin-rich protrusions at the front of migrating PGCs.

Altogether, these data indicate that SGPs release active Hh via the Hmgrc pathway. Hh directly binds to the Ptc receptor on PGCs. This binding leads to the internalization of the Hh-Ptc complex and activation of downstream signaling. Consequently, Smo relocalizes to the plasma membrane at the front of migrating PGCs, guiding their movement toward the Hh source.

## Hedgehog signaling directs cell movement through Tre1

An important question is whether conventional events downstream of Hh signaling (e.g. Ci-regulated transcriptional activation) are activated in germ cells and whether these change cell behavior. Current data support a non-conventional signaling model with a well-studied G protein-coupled receptor (GPCR) at its center. Multiple labs have previously shown the requirement for the GPCR *Trapped in endoderm* (*Tre1*) for PGC navigation through the midgut endoderm (Kamps et al., 2010; Kim et al., 2021; Kunwar et al., 2003, 2008; LeBlanc and Lehmann, 2017; Lin et al., 2020) (Table 1). *Tre1* mRNA is observed in the posterior pole plasm associated with germ cells even before PGCs are formed and persists through the early stages of migration, indicating that *Tre1* is expressed in the right place and time to be a candidate receptor to direct migration (Kim et al., 2021; Kunwar et al., 2003). Complete maternal loss of *Tre1* leads to severe defects in PGC navigation, with large numbers of PGCs failing to reach the gonads in more than 90% of mutant embryos (Kim et al., 2021), similar to the phenotypes observed with loss of *Hmgcr*. Early studies had suggested that Tre1 is specifically required for PGCs to escape the posterior endoderm pocket – hence the name ‘Trapped in Endoderm’ (Kunwar et al., 2003). However, more recent work from the same group (Lin et al., 2020), as well as studies from Kim, Hanlon and colleagues (Kim et al., 2021), suggest that this is not the case and support a model wherein the PGC defects observed in *Tre1* mutants are due entirely to a failure to sense and respond to guidance cues from very early stages (i.e. failed navigation). Altogether, these data implicate Tre1 in cell-autonomous PGC migration.

## Hh activation of Tre1 harnesses an intrinsic polarizing activity of PGCs for navigation

Several laboratories have demonstrated the presence of F-actin protrusions at the front of migrating PGCs beginning prior to their journey from the midgut pocket to their coalescence with SGPs (Jaglarz and Howard, 1995; Kim et al., 2021; Lin et al., 2020; Seifert and Lehmann, 2012). These protrusions have been observed in both fixed and living samples using a variety of actin-specific probes. Lin et al. (2020) have further demonstrated the presence of F-actin and Myosin II (MyoII) at the rear of migrating PGCs, consistent with the classic four-step model described for mesenchymal cell migration (Bodor et al., 2020; Innocenti, 2018). This model begins with formation of an actin-rich protrusion at the leading edge, followed by adhesion to a substrate, contraction of the cell body (aided by MyoII), and retraction of the rear edge. The classic model posits that cells are polarized from front to back in response to external cues, with the cellular machinery oriented accordingly: actin-driven protrusions at the front and myosin-driven contractions at the rear.

Lin and colleagues have proposed a PGC migration model that is more ameoboid-like (Liu et al., 2015; Paluch et al., 2016) and driven by rearward actin flows and active MyoII at the rear of the cell (Liu et al., 2015; Paluch et al., 2016). Importantly, Lin et al. (2022) have shown that PGCs have the intrinsic ability to generate actin flows and localize MyoII in the complete absence of directional information, as occurs when the PGCs are taken out of the embryo and cultured in a dish, or when PGCs are missing function of Tre1 – the key GPCR expressed and required for PGC navigation (Lin et al., 2022). However, PGCs require more than the simple ability to migrate: their functionality depends on their ability to migrate to a specific destination. In *Tre1* mutants, MyoII still polarizes in individual PGCs but is reduced in intensity, is more transient, and is random with respect to the orientation of the midgut pocket. Therefore, Hh signaling, operating through Tre1, does not create movement ability itself, but instead provides the crucial directional information that aligns and stabilizes the cells' built-in directional system to ensure PGCs move toward the signal source. This explains why PGCs mutant for any step in Hh signaling are still fully capable of migrating but fail to navigate successfully to the gonads.

Altogether, these findings suggest a role for Hh signaling and Tre1 activation in defining the leading edge or front of migrating PGCs. In keeping with this role, not only does activated Smo localize to the F-actin protrusions at the front of migrating PGCs, but so does Tre1 (Kim et al., 2021). Through studies in S2 tissue culture cells, in which molecules are easier to track, Kim et al. (2021) have shown that plasma membrane localization of the Tre1 receptor depends on Hh signaling.

Kim et al. (2021) have further uncovered a pathway whereby localized activated Tre1 recruits an enzyme activity of which culminates in the activation of Arp2,3 and formation of a branched actin network at the leading edge of migrating PGCs. They have shown that Smo and Tre1 localize to the actin-rich protrusions at the front of migrating PGCs, and so does Phosphatidylinositol-4-phosphate 5 kinase (dPIP5K; also known as PIP5K59B), the enzyme that converts PtdIns(4)*P* to PtdIns(4,5)*P_2_* (Table 1). PtdIns(4,5)*P_2_* is important because it directly binds to and activates Wiskott–Aldrich Syndrome protein (WASp). Activated WASp promotes Arp2,3-mediated F-actin polymerization to create branched actin protrusions. This pathway thus provides a mechanism whereby Hh-dependent Tre1 activation promotes actin assembly at the leading edge of PGCs. In further support of this model, WASp Interacting Protein (WIP; also known as Vrp1), which binds activated WASp, also localizes to actin-rich protrusions at the migration front. Importantly, in *Tre1* null PGCs, dPIP5K, PtdIns(4,5)*P*_2_ and F-actin all fail to localize to protrusions and are instead distributed along the entire plasma membrane (Kim et al., 2021) (Table 1). Overall, these findings indicate that Tre1 functions with Smo downstream of Hh activation to generate the cytoskeletal changes at the migration front for directed PGC movement. Furthermore, activated Smo has been shown to bind PtdIns(4)*P* (Jiang and Jia, 2015), the substrate used by dPIP5K to generate PtdIns(4,5)*P*_2_. Thus, it is possible that activated Smo not only recruits Tre1 to the migration front but also brings in the substrate for the Tre1-associated dPIP5K enzyme. We propose that the Hh/Tre1-dependent localization of the branched actin network defines the front of the cell by localizing and harnessing the intrinsic polarizing actin flows to localize MyoII to the rear of migrating PGCs.

## Reviewing the evidence

The cumulative data supporting a role for Hh as an attractant for PGC navigation are compelling. Core Hh pathway components are required for PGC navigation, and these components localize in a manner consistent with SGPs producing an active Hh ligand that can travel over long distances to reach and signal to migrating PGCs. Importantly, loss of *hh* or of any of the components required for Hh function, release or transmission can suppress the effects of ectopic expression of Hmgcr in almost every case tested, suggesting that these molecules are all downstream of Hmgcr. The protein localization data further support a model wherein the active Hh ligand is bound and internalized by the Ptc receptor on the PGC membrane. Ptc-mediated Hh internalization activates downstream signaling components leading to localized accumulation of Smo at the plasma membrane. Smo activates and recruits the Tre1 GPCR to generate the actin-rich protrusions required for directed PGC migration. Altogether, this pathway meets the expectations outlined for the PGC guidance system: Hh is produced by SGPs. Hh protein reaches and is internalized by the PGCs. Hh receptors and downstream effectors are expressed and required in the PGCs. Through Smo recruitment of Tre1 to the migration front, Hh signaling is linked to the F-actin assembly at the front of these cells to provide for directional movement. While this Review focuses on attractant cues, PGCs are repulsed by signals that might also interact with this pathway as part of this model (Box 2).

Box 2. A hypothesis for balancing attraction with repulsion: the wunens The primordial germ cell (PGC) repulsive cue(s) depend(s) on two homologous integral membrane lipid phosphate phosphatases (LPPs): Wunen and Wunen-2 (Hanyu-Nakamura et al., 2004; Starz-Gaiano et al., 2001; Zhang et al., 1997). The *wun* and *wun2* genes are broadly expressed in regions of the embryo where PGCs normally do not migrate, including in the hindgut, the central nervous system (CNS) and the ventral posterior gut endoderm. Hindgut-expressed *wun* is important for keeping the PGCs in the midgut pocket and could also guide PGC exit through the gut endoderm. *wun* expression in the CNS functions in the bilateral sorting of the PGCs into two clusters after they traverse the posterior endoderm. *wun* expression in the ventral midgut endoderm and CNS directs PGCs towards the mesoderm (Renault et al., 2010). Importantly, ectopic expression of either *wun* or *wun2* in mesoderm blocks PGC migration and their association with the SGPs (Renault et al., 2010; Starz-Gaiano et al., 2001; Zhang et al., 1997). The Wunens have been proposed to internalize, dephosphorylate and inactivate an attractant (LeBlanc and Lehmann, 2017). Yet the LPPs are not channels (Ren et al., 2013). Instead, they are enzymes that dephosphorylate a membrane-embedded phospholipid, releasing a free phosphate and leaving a dephosphorylated or underphosphorylated lipid in place. Based on the known functions of the vertebrate LPPs, we propose that the Wunens could repulse PGCs by affecting Smo and/or Tre1 localization to the plasma membrane; they could equally well disrupt production and/or function of the lipid-modified Hedgehog attractant.

The one experiment that is inconsistent with Hh functioning downstream of Hmgcr to guide PGCs is the finding that ectopic expression of Hmgcr in the most-posterior embryonic segments can attract PGCs in both wild-type and *hh* null embryos (Kenwrick et al., 2019). This controversial finding is clearly an outlier in the face of all other evidence. Furthermore, it is inconsistent with the more recent study showing that the ability of Hmgcr to attract PGCs depends on expression of Hh in the same cells (Deshpande et al., 2023). Specifically, Deshpande and colleagues showed that mismigration induced by ectopic expression of *Hmgcr* in the nervous system (*elavGal4::UAS-hmgcr*) is significantly suppressed by the simultaneous expression of *hh-RNAi* in the same cells.

## Conclusion

In summary, the widely expressed and extensively studied morphogen Hh appears to act ‘noncanonically’ as an attractant for PGCs to carve a path to their destination, the coalescing gonad. The factors affecting germ cell migration appear to function in a single pathway that controls all stages of migration, thus explaining the similarities in phenotypes observed with loss and/or misexpression of the major defining players in this process: Hmgcr, Hh, Tre1 and the Wunens (Box 2). Direct tests of our proposed unifying model would be to determine where Hh, Smo, Tre1 and the downstream effectors that mediate directed cell movement are localized in the PGCs of embryos mutant for Hmgcr and the Wunens. The finding that key regulators of the guidance cues and the guidance cue itself are provided by zygotically encoded genes expressed in the SGPs, and that the guidance response elements are provided by maternally encoded genes expressed in the germline, underscores the sophistication of the process. Orchestrating the events by which the two relevant cell types, which form at different times at distant sites under the control of factors produced in different generations, meet up, coalesce and form functional gonads is crucial for the species to persist.
